# Bringing Ecology Back: How Can the Chemistry of Indirect Plant Defenses Against Herbivory Be Manipulated to Improve Pest Management?

**DOI:** 10.3389/fpls.2018.01436

**Published:** 2018-09-27

**Authors:** Michael J. Furlong, Gurion C. K. Ang, Rehan Silva, Myron P. Zalucki

**Affiliations:** School of Biological Sciences, The University of Queensland, St Lucia, QLD, Australia

**Keywords:** herbivore induced plant volatiles, jasmonic acid, salicylic acid, indirect defense, parasitoid, chemical ecology, integrated pest management

## Abstract

Research on insect–plant interactions has highlighted the intricacies of constitutive and induced plant defenses. Of particular interest has been the relationship of natural enemies (especially parasitic hymenoptera) to herbivore induced changes to plants, especially their responses to herbivore induced plant volatiles (HIPVs). In recent decades this has been a fertile area for research, with elegant experiments showing that HIPVs are important in attracting natural enemies to plants. We critically appraise the application of work on HIPVs in plant–insect–natural enemy interactions. The promise of applications to improve pest management has not been forthcoming. We attribute this to a failure to include the multifaceted aspects of natural enemy–prey interactions – attraction, location, subjugation and experience. Attraction in an olfactometer by naïve parasitoids has not been translated to methodologically sound field-based estimates of higher parasitism rates. We highlight what needs to be done to better understand the information that HIPVs convey, how this is utilized by parasitoids and how a greater understanding of these interactions might lead to the development of new strategies so that this knowledge can be effectively deployed for improved pest management.

## Introduction

A wide range of pathogens and sucking, chewing and boring herbivores assail all parts of plants in nature. Recent research has revealed the intricacies and sophistication of a Pandora’s box of chemical interactions that mediate plant responses to this attack (see Kaplan, 2012; Schuman and Baldwin, 2016). Plants have a range of constitutive (e.g., Zalucki et al., 2001; Steppuhn et al., 2004) and induced chemical defenses (Kessler and Baldwin, 2002) that can act either directly or indirectly on herbivores. Induced defenses represent a sophisticated, layered set of responses that are modulated by a complex phytohormone system (Wu and Baldwin, 2010; Schuman and Baldwin, 2016; Howe et al., 2018). For example, necrotrophic microbes, some phloem-feeding insects and chewing herbivores induce the jasmonic acid (JA) pathway (Howe and Jander, 2008), whereas some phloem-feeding insects and biotrophic pathogens induce the salicylic acid (SA) pathway (Spoel et al., 2007). Cross-talk between these pathways, enables plants to regulate their defense responses according to the type of attacker (Koornneef and Pieterse, 2008; Thaler et al., 2012).

Following plant damage, induced direct defenses lead to localized and systemic elevation of toxic secondary compounds above constitutive levels, which affect the preference, performance and feeding behavior of herbivores (Wittstock and Gershenzon, 2002; Perkins et al., 2013; Zalucki et al., 2017). The increased release of volatiles from a plant after attack by a herbivore is considered to be an induced indirect defenze, communicating the location of herbivores on infested plants to parasitoids and predators of the attackers (Vet and Dicke, 1992; Dicke et al., 2003a,b; Turlings and Erb, 2018). Numerous studies have shown the importance of multi–trophic relationships in plant-insect-natural enemy “attack-defenze” systems. These may be as a result of aboveground (van Dam et al., 2003) or belowground herbivory (van Dam and Oomen, 2008; Qiu et al., 2009) and they can interact in complex ways (Soler et al., 2005, 2007; van Dam and Heil, 2011).

In natural environments it is very likely that plants suffer sequential and/ or simultaneous attack by numerous herbivores and pathogens. Plant pathogens can suppress the host plant defenses that they induce by leveraging SA-JA crosstalk using chemical toxins and the virulent effector proteins they secret (Xin and He, 2013; Kaloshian and Walling, 2016). In this context, the distinction between plant pathogens and insect symbionts can become blurred. For example, co-infection of tobacco plants with a begomovirus and its betasatellite represses JA-regulated defenses, allowing whitefly populations to increase (Zhang et al., 2012). In this system the compatibility of the tobacco–begomovirus interaction means that SA levels are unaffected, and the suppression of the JA-regulated defenses is independent of SA (Zhang et al., 2012). Similarly, herbivore manipulation of plant defenses has been associated with the chemical compounds and specific proteins in the saliva of chewing and phloem-feeding insect herbivores (Will et al., 2007; Elzinga et al., 2014; Villarroel et al., 2016), making the precise outcome of interactions difficult to predict. Thus, in any given system, simultaneous interactions, each of which can have positive or negative effects of varying degrees of magnitude at the individual and population level, likely manifest as an indeterminate orgy of multipartite interactions. This, coupled with an incomplete understanding of specific relationships, makes reliable predictions of outcomes problematic. This at least partly accounts for the near absence of successful field applications of technologies based on the deployment of herbivore induced plant volatiles (HIPVs) or the chemical manipulation of plants to suppress herbivore pest populations (Turlings and Erb, 2018).

## Exploiting Host Plant Odors for Pest Suppression

This is not to suggest that host plant odors have not been successfully manipulated for improved pest management. They provide the mechanism underpinning push- pull strategies (Cook et al., 2007), which rely on plant odors to repel pest insects from crops and attract them to non-crop plants at the margins of fields. The most prominent example of the successful deployment of this strategy involves the companion cropping of maize with the forage grass *Melinis minutiflora* or the forage legume *Desmodium* sp. and the planting of other grasses, e.g., *Pennisetum purpureum* or *Sorghum vulgare sudanaense* at the field margins (Pickett et al., 2014; Pickett and Khan, 2016). Volatiles produced by *M. minutiflora* or *Desmodium* sp. repel ovipositing stem borers from the maize crop (Khan et al., 2000), thereby providing the “push,” while other volatiles produced by the specific forage grasses planted on the margins attract the ovipositing females, thereby producing the “pull” away from the crop (Khan et al., 2000). This strategy, and well researched variants, has been adopted by approximately 120,000 small holders in sub-Saharan East Africa (Pickett and Khan, 2016). Despite this undoubted success, further claims that the attractive properties of volatiles emitted by *M. minutiflora* themselves result in meaningful greater parasitism of stem borers on maize (Khan et al., 1997; Midega et al., 2009) need to be treated with caution. These compounds clearly invoke strong behavioral responses in parasitoids in the laboratory (Khan et al., 1997; Tamiru et al., 2012), but their impact on pest mortality in the field requires further investigation. Reported parasitism rates are typically derived from small or unreported sample sizes and, even when statistically significant changes in parasitism rates are reported, increases are small and not contextualized with respect to other mortality factors (see Van Driesche et al., 1991). Similarly, although stemborer oviposition on maize has been demonstrated to increase attraction of egg and larval parasitoids in the laboratory (Tamiru et al., 2011), evidence demonstrating increased egg mortality in the field as a result is lacking. Further, the interpretation of increased larval parasitoid attraction as early recruitment of natural enemies in anticipation of egg hatching (Tamiru et al., 2011) is teleological. Indeed, *Cotesia sesamiae* (Cameron) (Hymenoptera: Braconidae) attacks late larvae of *Chilo partellus* (Swinhoe) (Lepidoptera: Crambidae) (Chinwada et al., 2003) and naïve females of this species are attracted to, but do not discriminate between, maize plants infested by *C. partellus* and the non-host *Busseola fusca* (Fuller) (Lepidoptera: Noctuidae) (Ngi-Song et al., 1995, 1996).

## Parasitoid Responses to Herbivore Induced Plant Volatiles

**FIGURE 1:**
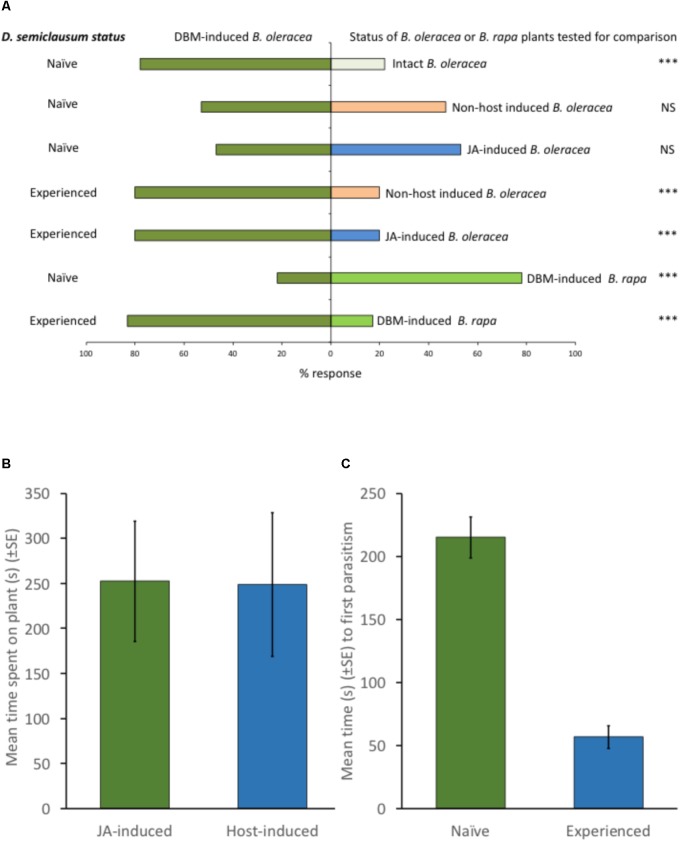
**(A)** Effect of previous experience on the responses of *Diadegma semiclausum* Hellén (Hymenoptera: Ichneumonidae) to cabbage (*Brassica oleracea* capitata cv sugarloaf) and Chinese cabbage (*Brassica rapa* pekinensis cv Wombok) host plants (6-leaf stage) in olfactometer bioassays. *Methods:* The olfactometer consisted of a glass Y-tube (0.8 cm internal diameter, 7 cm stem) with two 9.5 cm arms at a 60° angle leading to sealed glass chambers, each containing a single plant. Clean air (filtered through activated charcoal filters before entry into the apparatus) was drawn through the system with a vacuum pump at a rate of 1 L min^-1^. Test insects were introduced to the end of the Y-tube stem 6 cm away from the Y-split. A choice was considered made once a test insect breached the 2.5 cm mark up an arm of the Y-tube. Each test insect was given up to 10 min to respond, and each insect was used only once. For each pairwise combination of plant treatments, fresh female parasitoids were used until 30 individuals had responded. The Y-tube was rotated between each replicate, and between every five consecutive replicates, odor sources were replaced, and all glassware was washed in 95% ethanol, rinsed with distilled water and then dried at 75°C for 1 h. All tests were performed in a draught-free room at 24 ± 1°C under an artificial light. *Statistical tests:* Preferences of parasitoids in the various pairwise tests were analyzed using a *X*^2^-test. ^NS^*P* > 0.05 (*X*^2^_(_*_df_*_=__1)_ < 3.841); ^∗∗∗^*P* < 0.001 (*X*^2^_(_*_df_*_=1)_> 10.828). *Results:* Naïve parasitoids [= parasitoids (1–2 days post-eclosion)] that had not foraged for or oviposited into a host [*Plutella xylostella* L. (Lepidoptera: Plutellidae) ( = diamondback moth, DBM)] larva on a cabbage plant were more attracted to host-damaged plants (feeding by 10 early 3rd instar DBM larvae for 24 h immediately prior to test, larvae removed prior to olfactometer bioassay) than to*(intact plants (*P* < 0.001), but did not discriminate between DBM-damaged plants and non-host [*Crocidolomia pavonana* (F) (Lepidoptera: Crambidae) ( = large cabbage moth, LCM)] damaged plants (feeding by 10 2nd instar LCM larvae for 24 h immediately prior to test, larvae removed prior to olfactometer bioassay) (*P* > 0.05) or DBM-damaged plants and plants treated with jasmonic acid (JA) (aqueous solution of 0.1 μmolL^-1^ JA applied to plants 24 h prior to test; see Lu et al., 2004 for methods). Experienced parasitoids (= parasitoids that had oviposited into a host DBM larva on a cabbage plant) were more attracted to DBM-damaged plants than LCM-damaged plants (*P* < 0.001) or plants treated with JA (*P* < 0.001). Although naïve parasitoids were more attracted to DBM-damaged Chinese cabbage plants than DBM-damaged cabbage plants (*P* < 0.001), parasitoids previously experienced on DBM on cabbage plants were more attracted to DBM-damaged cabbage plants than DBM-damaged Chinese cabbage plants in subsequent olfactometer assays (*P* < 0.001). **(B)** Effect of DBM larvae (damage and presence) and jasmonic acid (JA) treatment on *D. semiclausum* post-alighting residency time on cabbage plants. *Methods:* Experiments were conducted in a wind tunnel with a Perspex flight chamber (160 cm × 65 cm × 65 cm). A fan circulated clean air (passed over an activated charcoal filter) through the chamber at 0.7 ms^-1^. Laminar airflow was obtained by passing air through a honeycomb of soda straws and a fine stainless-steel screen (1.25 mm aperture) before it entered the chamber. A single potted cabbage test plant (6-leaf stage) was placed in the centre of the chamber 45 cm from the screen and a single female *D. semiclausum* released 50 cm downwind. The responses of parasitoids to JA-treated plants (aqueous solution of 0.1 μmolL^-1^ JA applied to plants 24 h prior to test; see Lu et al., 2004 for methods) and to DBM-infested (10 early 3rd instar larvae for 24 h) plants were compared by alternating JA-treated and DBM-infested plants in the chamber and recording the *D. semiclausum* response; each parasitoid was given up to 5 min to respond, and each insect was used only once. When a parasitoid alighted on the test plant it was watched carefully and the time it spent on the plant (= residency time) recorded. The responses of single parasitoids to 9 plants of each treatment were recorded. *Statistical tests:* Data were tested for normality (D’Agostino and Pearson test) and then subject to an unpaired *t*-test (Prism 7, Graphpad Software, Inc., 2017). *Results:* Parasitoids spent as long on JA-treated plants (*n* = 9) as they did on DBM-infested (10 early 3rd instar larvae) plants (*n* = 9) [*t* = 0.036 (*df* = 16), *P* = 0.972]. **(C)** Effect of host experience on post-alighting parasitoid foraging efficiency. *Methods:* Single *D. semiclausum* cocoons were transferred to clean glass tubes (0.8 cm × 4 cm). Upon eclosion female parasitoids were fed (10% honey solution) and some parasitoids were then experienced by allowing a single wasp to forage on cabbage plants (6-leaf stage) infested with third instar DBM larvae in a mesh cage (45 cm × 45 cm × 45 cm). Once a parasitoid attacked a host larva it was removed and held in a labeled glass tube. Cabbage plants (6-leaf stage; *n* = 20) were infested with 20 late instar larvae and transferred singly to mesh cages for 24 h. A single naïve or a single experienced *D. semclausum* was then introduced to each cage, carefully observed and the time taken to attack the first host larva was recorded. *Statistical tests:* Data were tested for normality (D’Agostino and Pearson test) and then subject to an unpaired *t*-test (Prism 7, Graphpad Software, Inc., 2017). *Results:* Naïve parasitoids (*n* = 10) took significantly longer to locate and parasitize a larva after alighting on a DBM-infested cabbage plant than previously experienced parasitoids (*n* = 10) [*t* = 7.834 (*df* = 18), *P* < 0.0001].)*

**FIGURE 2:**
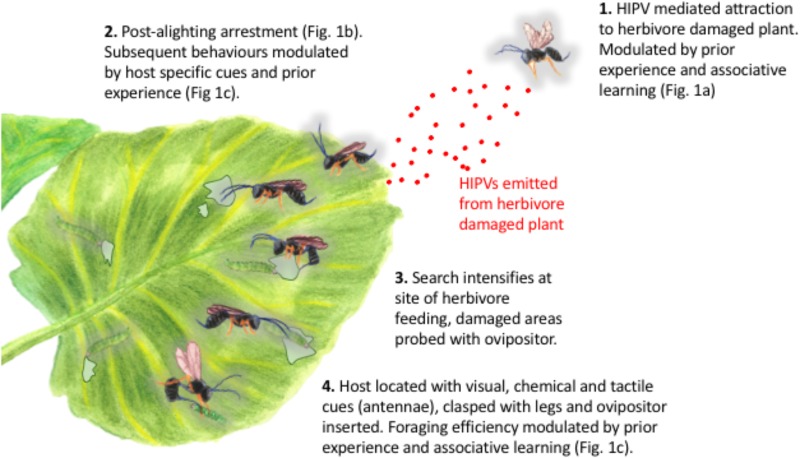
Attraction, orientation and post-alighting behavior of parasitoid wasps in response to host-herbivore infested plants. Post-alighting behavioral sequence based on *Diadegma semiclausum* Hellén (Hymenoptera: Ichneumonidae) as described by Wang and Keller (2002).

Although naïve parasitoids can be attracted to the volatiles produced by plants that are attacked by their host herbivores, our work shows that they can equally be attracted to volatiles emitted by those plants when they are attacked by non-host herbivores, or even when they are induced by a chemical elicitor, such as exogenous JA (**Figure 1A**). These responses are typically modulated by parasitoid experience, such that experienced parasitoids orientate preferentially toward plants upon which they have previously successfully located and parasitized a host (**Figure 1A**). Thus, although parasitoids might exhibit an innate preference for the volatiles produced by certain plants (Allison and Hare, 2009), such preferences can be overridden by experience (**Figure 1A**). Parasitoid orientation to a plant emitting volatiles that might be associated with the presence of host larvae is merely the first step in host location. Upon alighting on the plant surface, parasitoids typically become arrested (**Figures 1B, 2**), before increasing activity following the perception of post-alighting cues (which might include volatiles released as a result of herbivore feeding damage, non-volatile plant metabolites or herbivore frass), intensifying their search around sites of herbivore feeding damage before locating, sampling and then parasitizing their hosts (Wang and Keller, 2002; **Figures 1C, 2**). The vast majority of studies that seek to manipulate the responses of plants to herbivory in order increase biological control of pests rely on the application of a plant defense response elicitor (e.g., JA, SA or their derivatives) with the express purpose of attracting parasitoids to treated plants. The importance of subsequent parasitoid behaviors (see **Figure 2** for description of post-alighting fraging behavior) is then not considered, ignoring factors that are critical to successful host location and subsequent attack. For example, although the diamondback moth parasitoid, *Diadegma semiclausum* (Hellén) (Hymenoptera: Ichneumonidae), orientates toward and alights on intact, host-free cabbage plants treated with exogenous applications of JA, its post-alighting behavior is inhibited. Our work shows that the residency time of individuals alighting on JA-treated plants is the same as that of individuals landing and foraging on plants infested with host larvae (**Figure 1B**). Furthermore, experienced individuals locate hosts much more quickly than naïve parasitoids, demonstrating the importance of post-alighting cues in successful host location (**Figure 1C**). The lack of consideration of the subtlety and sophistication of the post-alighting chemical ecology of host–parasitoid interactions likely contributes to the dearth of examples where these strategies have been successfully deployed in the field. Further research to better understand these behaviors in parasitoids that are targeted for field manipulation by exploiting host–plant interactions for improved pest management are encouraged.

Other examples of “field” experiments that demonstrate changes in parasitoid attack rates are small-scale and typically lack adequate controls. Invariably the design consists of a single field into which “test” plants are placed to assess parasitism, usually with both treatments juxtaposed (Lou et al., 2005, 2006; de Lange et al., 2018). Apart from the issues of pseudoreplication associated with such an approach, the key problem is that any change in parasitism rate reflects either a change in parasitoid searching efficiency or a change in number of parasitoids foraging due to a combination of short (within field) or longer-range attraction. If the mechanism responsible for increased parasitism is an increase in parasitoid foraging efficiency then the lower parasitism of non-treatment plants within the experimental field reflects the confounding influence of the more attractive plants, which results in decreased attacks on non-treatment plants within the field. What is required is the deployment of independent control blocks, without treatment, that allow background parasitism rates to be assessed and then compared with parasitism rates in independent treated blocks. Without this approach, the apparent changes in parasitism in treated plants cannot be quantified in appropriate context as such changes clearly do not necessarily reflect increased parasitism at the population scale, which must be the goal for improved pest management. The use of multiple fields (blocks) with treatments in a randomized design is a better approach but even when this is done, experimental units are small, e.g., 7 × 7 plants (Poelman et al., 2009) or 4–10 plants (Simpson et al., 2011) and not independent. Although such experiments can provide important and useful preliminary information, their relevance to actual farm fields is limited, except perhaps in situations where fields are very small, and they require larger scale follow-up studies.

## Utilizing Herbivore Induced Plant Volatiles for Improved Biological Control and Pest Management

Herbivore induced plant volatiles can be used by parasitoids and predators to locate plants upon which their arthropod hosts or prey are feeding (e.g., Reddy, 2002; Sobhy et al., 2014). However, the role of these compounds in effecting arthropod herbivore population suppression at the field scale is still to be demonstrated. Synthetic volatiles have been deployed as lures within crops (Rodriguez-Saona et al., 2011), and although some success has been demonstrated with respect to the attraction of arthropod predators and parasitoids (James, 2003; James and Grasswitz, 2005), other studies show that HIPVs can have repellent effects on natural enemies (Braasch et al., 2012). The scale over which parasitoids and predators can be manipulated by synthetic HIPVs is typically small (<10 m) (Lee, 2010; Braasch and Kaplan, 2012), meaning that the major effect of their deployment is likely to be the localized redistribution of natural enemies within crops. This has been shown for some parasitic hymenoptera where the attraction to lures affects local abundances by depleting parasitoid numbers elsewhere in the crop (Braasch and Kaplan, 2012). The redistribution and retention of natural enemies in areas of the crop from which their prey might be absent (**Figure 1B**), and where their foraging behavior is further disrupted by the absence of appropriate post-alighting cues (**Figure 2**), is likely to disrupt rather than facilitate the conservation biological control strategies into which HIPVs are integrated. Given these effects perhaps the ways in which HIPVs can be best deployed for improved pest management should be re-evaluated; rather than attractants to support conservation biological control, they might be best utilized as arrestants to complement augmentative or even inundative releases of natural enemies (Kaplan, 2012).

The introduction of synthetic HIPVs into crops can induce natural defense responses in nearby plants (Kaplan, 2012) and the application of chemical elicitors (Sobhy et al., 2014) or the genetic manipulation of plants (Beale et al., 2006) to make then more attractive to natural enemies has been advocated. Various elicitors have been shown to change the volatile profiles of maize plants infested by *Spodoptera littoralis* (Boisduval) (Lepidoptera: Noctuidae), such that they become more attractive to parasitic hymenoptera (Rostas and Turlings, 2008; Sobhy et al., 2014) in laboratory assays. When the strategy was field tested, despite the elicitors changing plant volatile profiles in ways consistent with previous work in the laboratory, reliable increases in parasitism of *Spodoptera frugiperda* (J.E. Smith) could not be demonstrated (von Mérey et al., 2012). Similarly, genetic manipulation of *Arabidopsis thaliana* and wheat plants so that they constitutively produced aphid alarm pheromone was successful at repelling aphids and attracting aphid parasitoids in the laboratory (Beale et al., 2006; Bruce et al., 2015). However, when tested in the field wheat plants became infested with aphids and these showed no increased levels of parasitism (Bruce et al., 2015). In addition to changing the responses of parasitoids to induced plants, HIPVs also affect herbivore–plant interactions. In the *Brassica oleracea*–*Plutella xylostella* interaction, herbivore and JA-induced plants are more attractive to ovipositing *P. xylostella* (Lu et al., 2004), *D. semiclausum* (**Figure 1**) and predatory lacewing larvae (Furlong, unpublished data). The increased attraction is temporary, and plants revert to their original level of attractiveness 3–4 days after the application of JA or a feeding event (Ang et al., 2016). Selective application of JA to *B. oleracea* plants in the field can manipulate the spatial patterns of *P. xylostella* oviposition (Ang, 2018), raising the possibility of utilizing JA to temporarily induce parts of the crop as a “temporal trap-crop,” where *P. xylostella* eggs can be concentrated for destruction.

## Conclusion

Understanding of the molecular basis of plant responses to herbivore attack has increased markedly in recent years, but the technologies that have been developed have not led to more effective use of natural enemies for pest management. This is at least in part due to an under appreciation of how natural enemies utilize and respond to the cues they perceive from herbivore damaged plants under field conditions. Simply deploying HIPVS to attract natural enemies has not worked, however, developing a better understanding of the ecology of these interactions will inform how HIPVs and other components of plant–herbivore–natural enemy interactions might be more effectively used in as yet untested ways.

## Author Contributions

MF and MZ conceived and designed the overall study. GA, RS, and MF designed the experiments. GA and RS conducted the experiments. MF and MZ wrote the paper. GA did the artwork for **Figure 2**.

## Conflict of Interest Statement

The authors declare that the research was conducted in the absence of any commercial or financial relationships that could be construed as a potential conflict of interest.
